# A sustainable balance between innovation and risk: How the “right to science” affects China’s medical biotechnology regulatory policy

**DOI:** 10.1016/j.csbj.2024.04.027

**Published:** 2024-04-16

**Authors:** Yiping Han, Lindsay L. Fan, Yang Xue

**Affiliations:** a School of Social Sciences, University of Manchester, Manchester, UK; b North Oconee High School, Bogart, GA, USA; c Center for Biosafety Research and Strategy, Tianjin University, Tianjin, China; d Law School, Tianjin University, Tianjin, China

**Keywords:** Right to science, Medical biotechnology, China, Regulatory policy, Biosafety and biosecurity, Risk and innovation

## Abstract

Medical biotechnology is at the forefront of scientific progress, with humanity facing a critical juncture during the pandemic. However, to maximize these benefits, governments face the complex challenge of reconciling innovation and risk. A sustainable balance is critical, as extreme measures such as blanket bans on biotechnology research could hamper progress, while unfettered research could pose an existential threat. The need for effective regulation has become apparent in the context of recent controversies surrounding pharmaceutical biotechnology. Governments face the challenge of reconciling precaution with innovation, necessitating a dual strategy fostering both principles. This paper explores the delicate dynamics of innovation and risk in pharmaceutical biotechnology, focusing on the evolving landscape in Europe, the U.S., and, notably, China. At the same time, we delve into the regulatory landscape and examine the role of the “right to science” in shaping Chinese policy. This paper further applies the right to science that has received the interests of medical biotechnology regulatory policymakers: understanding the role of scientific claims in regulating emerging technologies and analyzing the impact of major regulations on the ability to sustainably balance innovation and risk. We believe that a comprehensive global effort is needed to harmonize these two principles, highlighting the imperative of responsible governance in steering the trajectory of this powerful scientific frontier. The insights gained from the Chinese experience offer valuable implications for global policymakers facing similar challenges.

## Graphical Abstract

**Figure:**
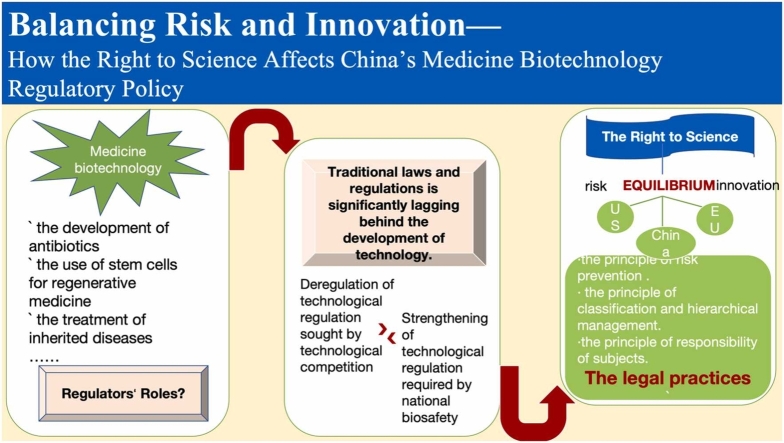

## 1 Introduction

Medical biotechnology has great potential to contribute to sustainable development, such as development of antibiotics, use of stem cells for regenerative medicine, treatment of genetic disorders, and control of vectors of dangerous human pathogens. Governments need to justify the inevitable choices that must be made to maximize the sustainability of the resulting development opportunities arising. Banning all biotechnology research, for example, might maximize human safety in the short term, but endanger it in the long term because medicine, agriculture and manufacturing are unable to innovate. Conversely, placing no restrictions on research may hasten development of life-saving products, but also increase the probability of existential damage to human civilization [1]. Medical biotechnology has advanced with remarkable speed and impact – hence both the need and demand for benefits, and the concern about risks. The recent gene-editing incident involving Jiankui He and the twin babies, [2] as well as U.S. deaths from CRISPR gene-editing therapy, [3] along with the respective follow-up handling, and in particular the recent calls for strengthened oversight of risky pathogens [4], have demonstrated a profound conflict between the urgent need for cutting-edge and disruptive medical biotechnological innovation, and the logic of prevention to inhibit risks. Regulators must balance divergent objectives: whether the principal aim of biotechnology regulatory policy is elimination of risk, or willingness to take acceptable risk based on the value of the opportunity [5]. The former is sometimes described as the precautionary principle, and the latter as the innovation principle [6]. Therefore, the relevant regulatory policies need to be both preventive and innovative, not only through construction of specific policies to deal with various types of uncertainties, but also through institutionalization of policies to encourage research and industrial innovation to ensure the vitality and innovation of biomedical research. This dual strategy is not without problems. “Obviously, governments thought that biotechnology was something worth developing and they supported it with alacrity. Yet they also present themselves as impartial overseers of what many see as a risky endeavor. This ambiguity later proved to be one of the sources of public distrust” [7]. Humanity is at a critical moment in the worldwide evolution of powerful experimental capabilities in the life sciences, motivated and humbled by an ongoing pandemic, faced with the possibility both of substantial harm and good from continued work on medical biotechnology. The 2018 gene-editing incident stirred considerable controversy [8]. Researchers implanted edited embryos without informing the subjects, ostensibly to cure diseases, notably immunizing against HIV [8]. Despite the claims of justifiability, ethical concerns emerged, including issues of informed consent and compromising human dignity by potentially contaminating the broader gene pool [9]. Consequently, the biologist faced punitive measures solely under criminal law, but no clear regulatory norms exist for project approval, operational procedures, or result evaluations [10]. China’s Constitution and laws primarily promote technological progress, yet this apparent encouragement of innovation is often aspirational and lacks detailed provisions [11], [12], [13]. Examining China’s legal framework for biomedical technology, text encouraging technological development can be gleaned from various legal documents, but when specific safety incidents occur, the legal system resorts to the most severe measures of state violence through criminal law [14]. This unclear stance appears to reflect a regulatory dilemma in China’s management of biomedical technology, navigating between promoting development and ensuring safety, and grappling with the dilemma of innovation versus risk [14]. Its directional inclination parallels precaution and innovation policy advocacy in the EU and U.S.; for instance, the EU has explored the extent of technological exploration considered safe and ethically permissible. [15]. The causal underpinnings of these policies in the EU and U.S., marked by debates on precaution and innovation, sheds light on the mechanisms steering this policy preference. This analytical framework might can be applied to the Chinese context, where similar choices confront regulators. Within the right to science framework, encompassing the rights of scientists and the public, we aim to assess the practical implementation of legal systems across diverse jurisdictions. With its dual focus, this right assumes prominence in deliberations between innovation and risk, providing a distinctive vantage point for nuanced analysis.

This paper first provides background information on risk in medical biotechnology and the hidden dynamics underlying its interplay with innovation. To develop long-term solutions to societal issues, Europe and the U.S. aim to optimize the balance between innovation and biosafety and biosecurity risk through ongoing improvements in policy and decision-making. Emerging biotechnologies are having ever more disruptive impacts on society; particular controversies and concerns have arisen from research on human germline gene editing and reproductive cloning. Article 10 of the *European Convention on Human Rights* (ECHR) and the draft American *Declaration on the Rights and Duties of Man* (American Declaration) have begun to take right to science issues into account, i.e., that research does not violate basic social morality, emphasizing that the benchmark of national restrictions on research is to ensure that citizens are not afflicted by fear. With the emergence of China as a major player in research over the past decade, new perspectives will emerge on the policy influence of the right to science. In the context of Regulatory policies for medical biotechnology in China are becoming increasingly important. To this end, we then focus on two questions: I) What is the “right to science?” In what way does the regulation of emerging technologies involve it? II) How have the major regulations and laws surrounding this topic helped in creating a sustainable balance between innovation and risk in medical biotechnology? We conclude with an analysis of the reasons why China has developed these regulatory policies integrating the right to science. China’s experiences will have useful implications for countries around the world.

## 2 Sustainably balancing innovation and risk

### 2.1 Risks of medical biotechnology

In recent years, with the increasing social demand for biomedicine, competition in biomedical technologies has become white-hot. Competition leads to wanton disregard for legal, ethical, and moral norms in research; rules are overstepped, including unauthorized clinical applications without rigorous verification of safety and efficacy, [16] contributing to social risk. Meanwhile, under economic impetus, the hidden power of capital behind this research harbors a strong desire to break through existing systemic limitations. The possibility of technological abuse and illegal acquisition of economic benefits has been increasing [17]. At the same time, because it takes a large amount of clinical data to validate the safety of biomedical technologies, the implementation of relevant governance rules normally requires a continuous cycle including data collection, risk assessment, and rule modification [18]. The scientific community can currently only assess the safety, ethical, and moral issues of technology based on assumptions about potential risks, leading to a dilemma in which development of regulatory systems such as traditional laws and regulations is significantly lagging technological development. In particular, when it comes to judicial determination of clinical medical applications in China, it is still difficult to effectively determine the legal causes and effects of biomedical technologies and what constitutes willful criminal intent, making it difficult to give full play to the deterrent effect of the law [8]. The uncertainty of technological risk is primarily reflected in the failure of checks and balances in traditional governance structures.

### 2.2 Why a sustainable balance between innovation and risk is needed

In China, the recent widespread social application of medical biotechnology has not only further fueled research competition within the scientific community, but also exacerbated polarization outside the scientific community about regulatory trade-offs between objectives [19]. In these circumstances, the contradiction between the deregulation in light of competition, and stronger regulation required by national biosafety (biosecurity) concerns, has gradually led to an unbridgeable rift between biotechnology regulation and technological competition policy. A possible conclusion is that innovation centered on technological advances is widely recognized as an essential risk-inducing factor in medical biotechnology.

First, the byproducts of innovation-oriented policies trigger technological risks. For decades, China has placed great emphasis on the biomedical industry and medical biotechnology, promoting innovation by generously supporting research and continuously exacerbating the impact of intense competition within the scientific community for breakthroughs. Some Chinese researchers are already motivated by their research interests, as well as economic ones, to falsify scientific data [20]. In addition, Chinese researchers have repeatedly crossed “red lines,” triggering intense international controversies [21], [22], [23]. In the face of the by-product of innovation-oriented policies - the unspoken rule of “judging heroes by their research and development” – the governance of Chinese medical biotechnology is in urgent need of reshaping.

Second, innovation-oriented research has reasonably raised public demand for good governance of technological risks. The rapid development of biomedical technology is attributed to the new strategies and approaches it provides for the diagnosis, treatment, and prevention of human diseases [24]. However, when the pursuit of innovations threatens to infringe upon basic human rights, such as safety [25], the right to informed consent [26], and possible permanent effects on future genetics, it raises social questions involving public safety, social equity, ethics, and morality. Even worse, under the current censorship system, scientists have the opportunity to bypass scrutiny by policymakers and the public by adopting a strategy of fait accompli for major scientific decisions involving the fate of certain human societies. This failure of oversight and lack of participation further exacerbates public insecurity and raises the need for good governance [27].

On the surface, this argument between innovation and risk originates from the inevitable conflict between rapid technological development and lags in risk regulation, but it is essentially a binary struggle between worry and control based on national interests, and competition and leadership. The solution is not merely to support regulatory policies when promoting development or to balance innovation while emphasizing prevention, but also to consider how both measures can be accommodated by institutional logic. Therefore, a sustainable balance between innovation and risk seems to be a timely adaptation to the general trend.

### 2.3 Medical biotechnology regulatory policies in the U.S. and Europe

Analyzing the legal biotechnology frameworks designed by the United States and the European Union (EU) regarding the right to science provides insights into the regulatory characteristics both two regions. The U.S. is exhibiting a more permissive and equivocal stance in the context of its competition in preparation of legal risk assessment standards. The EU, on the other hand, appears to have adopted a more cautious and conservative approach to new technologies, particularly about research freedom and the limitations associated with the right to science.

In the U.S., no judicial litigation directly pertinent to the current subject matter has emerged, and cases explicitly advocating for rights protection and redress in connection with the right to science have not been discerned. Although garnering insights from Supreme Court precedents on this matter may pose challenges, it is relatively straightforward to extract fundamental principles and perspectives from the text of the U.S. Constitution, which assures the freedom of expression [28]. As contended in later sections of this text, central to the right to science is the freedom to conduct research and benefit from its outcomes. This encompasses the entitlement of scientists or researchers to enjoy the fruits of their endeavors, including the liberty to actively participate, contingent upon ensuring equitable remuneration for the costs borne by pioneers in exploration. Furthermore, the First Amendment safeguards the freedom to disseminate scientific information. Consequently, the right to free expression partially realizes the right to science. In the absence of specific federal legislation addressing genome editing, regulatory oversight at the federal level seems predominantly vested in the authority of the Federal Drug Administration (FDA). The FDA’s engagement with genome editing is administered through its drug management protocols, rather than being expressly stipulated by existing regulations. Over time, the FDA has traditionally assumed responsibility for overseeing pharmaceuticals and medical equipment, encompassing biologics within its purview [29]. A potential lacuna in the U.S. approach toward biotechnology becomes apparent when scrutinizing the broader regulatory framework. While scientists may encounter challenges securing public funding, it appears that it is feasible to circumvent governmental intervention in funding through support from private sources. Furthermore, the regulatory oversight of the FDA over the distribution, utilization, and clinical trials of these entities might be compromised if the technology results in the creation of commercial products falling outside the categories of pharmaceuticals and medical devices. In the absence of public or governmental financing, supported by private financial sources, scientists and researchers might proceed with implantation of modified embryos into consenting and well-informed subjects. This process appears to have forged a shortcut.

Within the context of the European Union (EU), much attention is directed toward the *EU Charter on Fundamental Rights*, a pivotal document which dedicates an entire chapter to exploring the intersection of biotechnology and human rights [30]. At the same time, the *Charter* incorporates certain principled articles from the *Oviedo Convention* asserting the inherent and non-negotiable nature of human dignity. Nevertheless, neither the primary text of the *Charter* nor subsequent articles define or explicate the concept of “human dignity.” Technologies found to breach public order and ethical standards are ineligible for patent approval, as outlined in the 1998 *Biotech Directive* [31]. The 2014 *Clinical Trials Regulation* [32], specifically prohibits drugs capable of inducing changes in the germline, while endorsing clinical trials of somatic cell gene therapy products. The ban highlights the EU's concerns about potential interference with the reproductive germline of research subjects [32]. These regulations impose impediments on the legality of human germline genome editing, affecting both approval of patent applications and authorization of clinical trials for biomedical products. As a result, the EU has ruled out financial support at the project approval stage. This prohibition is evident from a close examination of Horizon 2020, an initiative set up by the EU to foster industry development, enhance competitiveness, and highlight the potential of technology [33]. Article 19 of the *Horizon 2020 Regulation* explicitly prohibits research involving modification of the human genome [33]. The term “heritable variations” is employed within the program to characterize research falling under this category, and financial support is withheld for studies involving embryos for commercial purposes. While there is no outright ban on research within the EU involving human somatic cells, funding for such efforts is nevertheless subject to more lenient rules, contingent on compliance with national laws.

When examining the factors contributing to these differences in biotechnology policies, one key factor is likely rooted in distinct public perceptions and ethnic considerations influencing policy preferences [19]. In Europe, government intervention in social life often appears more stringent than in the U.S., reflecting a higher acceptance of state regulatory measures [34]. Additionally, Europeans express heightened concerns about environmental issues, while Americans prioritize their personal lives over environmental matters and broader societal impacts of biomedicine beyond the private sphere [35]. Expanding on this ideological analysis, another important aspect to observe is the role of the political system in law formulation [36]. In both Europe and the U.S., the party system plays a crucial role in regulatory legal issues [37]. In the 1990s, a significant role reversal occurred in the European and American monitoring systems, coinciding with shifts in the performance of political parties in the two jurisdictions: the Green Party gained more seats in the EU, and the Democratic Party, with a preference for stricter risk control, gradually lost ground to the Republican Party [38]. An ongoing policy discourse persists between Europe and the U.S. regarding biomedical regulation, specifically revolving around the dichotomy between precautionary and innovative approaches. The European Community formally embraced the precautionary principle in 2000, prioritizing evaluation of scientifically uncertain risks that could potentially pose hazards to human safety or the environment, contradicting the trajectory of societal progress [39]. Although innovation has garnered theoretical and political support as a regulatory paradigm for technology within European political theory over the past decade, it has not emerged as an independent theoretical assertion divorced from precaution [40]. Expressed differently, in the legal discourse concerning regulation of science and technology, the efficacy of decision-making within the EU has been diminished in determining whether innovation or precaution is more aligned with public expectations. As revealed in our scrutiny of relevant legislative documents on the “right to research,” the EU continues to employ the precautionary principle, notwithstanding the recognition of innovation as a fundamental value.

Moreover, the EU has refrained from providing additional elucidation on reconciling the foundational differences between the two concepts [41]. Conversely, the lenient legislative posture of the U.S. regarding oversight of technology has not been a perennial norm. Since the 1990s, the EU has assumed the role previously held by the U.S. and currently stands as the foremost advocate for identifying risks in advance and serving as a protective barrier [42]. The EU and U.S. have adopted seemingly divergent positions concerning emerging technologies or, more broadly, legal issues related to technology, including formulation of legal frameworks concerning the right to science. China appears to adopt an intermediate regulatory approach, considering the regulatory experiences of both Europe and the U.S.

## 3 Application of the right to science on biotechnology regulatory policy

### 3.1 The right to science

In 1948, the Universal Declaration of Human Rights established the “right to science” [43]. The *International Covenant on Economic, Social, and Cultural Rights* (ICESCR) incorporates the “right to culture”, providing a more expansive framework for understanding the “right to science.” ICESCR affirms the right of individuals to participate in benefits arising from scientific and technical endeavors while safeguarding their research. Furthermore, it articulates the rights of the signatories to engage in research, emphasizing their obligation to furnish the resources and support necessary for the advancement of such efforts [44]. In 2012, Farida Shaheed was designated by the United Nations Human Rights Council to investigate the rights to science and culture [45]. The ensuing manuscript posited that the freedom to engage in research and investigations constitutes an integral facet of the right to actively partake in cultural pursuits. The right to science is posited as a constituent element of the right to culture, elucidating its nexus with societal culture by reaffirming the intrinsic correlation between scientific and cultural endeavors. Contrary to prevailing misconceptions, rights such as the freedom of association and the capacity to make informed decisions are not inherently incompatible [45]. The preceding analysis highlights that the right to culture, positioned as a subordinate concept within the realm of knowledge, along with the freedom to actively participate in and undertake activities associated with it, substantively underpins the intrinsic creative essence of both domains. Hence, contesting the assertion of an inherent connection between the right to knowledge and the liberty of creativity becomes a formidable task. Clarity regarding the specific nature of this freedom is imperative. International law safeguards the freedom inherent in research and various creative pursuits, a recognition explicitly articulated for the first time by ICESCR [44]. This freedom, derived from the *Universal Declaration of Human Rights* (UDHR), albeit without explicit articulation, is commonly construed to encompass the freedoms of expression, contemplation, and the opinions enunciated within UDHR [46]. Substantiating this assertion is the *European Convention on Human Rights*, which distinctly delineates the freedom to engage in scientific and artistic pursuits, predominantly attributing them to the rights associated with freedom of thought and expression [47].

Hence, the foundational tenets encapsulating the right to science is delineated as follows: each individual has the right to engage in science and avail themselves of scientific progress. This liberty encompasses both the freedom to conduct research – a facet primarily pertinent to scientists – and to reap the benefits emanating from that research. The latter freedom entails the right of scientists to the benefits of research (a domain intricately linked with intellectual property rights, though not expounded upon in detail herein for the sake of brevity), as well as the freedom of the broader public to derive benefits from research. Concurrently, the authors uphold the foundational tenet that any subordinate conceptual entities encompassed within the realm of knowledge must align with the inherent attributes of knowledge. This stance emanates from the belief that, as previously asserted, we construe science as a manifestation of knowledge, and assert that knowledge itself possesses an intrinsic, inviolable moral value. To adhere to this perspective, the application of knowledge must not contravene or compromise moral principles. Furthermore, the liberty to engage in research, akin to the freedoms associated with other human social activities, must operate exclusively within democratic legal frameworks and must not transgress fundamental ethical precepts [47].

### 3.2 Application of the right to science

The *Universal Declaration on the Human Genome and Human Rights*, which was adopted by UNESCO in 1997, is frequently regarded as the initial text to receive significant attention. The *Declaration* elucidates several fundamental ideas, such as those outlined in Articles 1–4, which highlight the paramount importance of safeguarding the rights of research subjects, particularly about the preservation of their confidentiality and the assurance of their right to informed consent [48]. The right to science relates to the imperative to ensure that genetic research upholds human dignity and respects the dignity of all individuals, as well as the need for transparency in disclosing the technical benefits of human genome research to the general public. The *Universal Declaration on Bioethics and Human Rights*, adopted in 2005, primarily outlines a distinct set of ethical principles governing the application of biotechnology in a human context. These principles encompass a broad spectrum of values, including the right to science, ranging from the right to engage in science to the rights to informed consent and privacy. Article 3 underscores the significance of upholding human dignity and freedom; Article 2 further underscores the constraints on exercise of scientific freedom [49]. We believe that the ultimate expression of the right to science in emerging science and technology regulation is not to provide generalized advice on one’s dealings with science and technology, but instead to provide counsel in related debates and decision-making processes by explaining the context of the relevant activities and clarifying the policy-oriented controversies in complex socio-technical systems. The right to science is legally reflected as the “hard” institutional system regulating the behavior of actors involved in science and technology. The regulatory balance between prevention of risks and innovation involves two competing objectives: intrinsic freedoms based on the inviolability of the right to science, and the principles of morality. These are particularly relevant as the impact of emerging biotechnologies on human society becomes increasingly disruptive. With the controversies and concerns arising from research on medical biotechnologies such as human germline gene editing and human reproductive cloning, this policy trade-off has gradually been taken into consideration within legal frameworks and has been set up as a legal no-go area by numerous countries around the world, setting up a legal “red line” concerning the value of rights. The governance of Emerging Science and Technology (EST) is frequently balanced in various ways with more definitive governance models [50].

This balancing act makes use of the exclusive role of the right to science. On the one hand, governance incorporating reflection on the right to science is not merely a matter of compliance with laws and regulations – which is necessary, but not sufficient, to sustainably steer emerging technologies in a better direction. In the face of uncertainty, anticipatory assessment of the impact of emerging technologies on scientific validity must be a priority. The result is governance which rethinks the right to science, emphasizing not only the need for emerging technologies to be guided by a given principle or norm, but also, to some extent, for the right to science itself to evolve alongside emerging technologies. On the other hand, governance which rethinks the right to science is in general more relevant to how right to science issues are constructed than to the outcome of relevant assessments. Therefore, it is concerned with the institutional conditions that the procedures for assessing individual projects must satisfy, focusing on selection of procedures based on their contextual relevance to actors, and the value dimension of their judgment in determining the meaning and scope of the norms. The result is a sustainable balance based on rules, context, and values [51].

## 4 Impact of the “right to science” on China’s medical biotechnology regulation

China, having ratified ICESCR, stipulates that only rights acknowledged and endorsed by its Constitution or domestic legislation are recognized under law. Despite the clear mandate for human rights in the Constitution, the right to science is not explicitly articulated. Article 47 guarantees citizens the freedom to conduct research, yet it neither addresses limitations on this freedom, nor extends its purview to encompass the enjoyment of scientific discoveries [13]. Conversely, the principles and particular legislative practice of regulation of medical biotechnology in China embody the right to science, the inviolable intrinsic freedom of scientific research, and the principle of not violating or undermining morality in legal policies pertaining to regulation of medical biotechnology.

### 4.1 Impact on regulatory principles

Several principles in Chinese regulation are however based on the right to science (See below Table 1). First, the principles of biosafety and biosecurity risk prevention that it upholds are consistent with the biotechnology regulation idea of “safe enough” implemented in Europe: i.e., uncertainty alone cannot be used as a reason to postpone the adoption of preventive measures; whoever develops a technology bears the responsibility and obligation of proving its harmlessness. If a technology holds a possible risk that has not yet gained scientific consensus, it must be regulated in a precautionary and prudent manner [52]. The advantage of the precautionary principle is that it cautiously grasps current controversial research directions and carefully examines the possible social risks of technologies, such as threatening life and health, violating human dignity, and impacting traditional culture and ethics, and minimizes the harms, avoiding having to remedy the problem after the fact due to over-consideration of the prospects and economic benefits of application. As a result, biomedical practitioners are required to strengthen risk control of their research and implementation, conduct adequate risk assessments and feasibility demonstrations, formulate risk prevention and contingency plans, and effectively supervise the entire process.

**Table 1:** Impact on the regulatory principles of the “right to science” on China’s medical biotechnology policy.

Impact of the “Right to Science” on China’s Medical Biotechnology Policy		
	Freedoms supported by the "right to science"	Limitations set by the "right to science"
**Impact of Regulatory Principles**		
*Principle of risk prevention*	**It puts almost its efforts into setting the limitation of freedom based on the “right to science”:** ·Technological uncertainty cannot be used as an excuse ·Whoever develops a technology bears the responsibility of proving that it is harmless ·Risk control of scientific research projects and implementation process ·Risk assessment and feasibility demonstration of scientific research and its results ·Risk prevention and contingency plans	
*Principle of categorized and hierarchical management*	**It aims to harness the contradictions between freedom and its limitations in the context of the "right to science”** : • Categorized and hierarchical management: high-, medium-, and low-risk • A catalog of new biomedical technologies in terms of risk level • The possible ethical and moral risks of biomedical technology • An open strategy of dynamic adjustment	
*Principle of responsibility of subjects*	• Adhering to the principle of bold research and prudent promotion	• Increasing scientists’ awareness of safety responsibility • Ethical self-discipline • Legal responsibility of medical institutions • A system of rewards and punishments

Second, the principles of classification and hierarchical management are currently a common choice in the field of international biotechnology security governance. In China, Chapter 4 of the *Biosafety Law*, entitled “Safety of Biotechnology Research, Development and Application,” states that “the State implements categorized management of biotechnology research and development activities,” and categorizes biotechnology R&D into three categories according to the degree of risk to public health, industrial agriculture and the environment: high-risk, medium-risk, and low-risk. The *Regulations on the Administration of Clinical Application of New Biomedical Technologies (Draft for Public Opinion)* implements hierarchical management of new biomedical technologies in clinical research, including medium-low- and high-risk, and calls for development of a catalogue of new biomedical technologies by risk level [53]. It should be noted that as intentional misuse of biotechnology is a low-probability, high-consequence potential event, it requires potential risk as a key consideration in risk assessment, and thus relies heavily on the subjective judgment of expert knowledge and is affected by individual expert knowledge, experience, and foresight. This not only requires practitioners to adhere to a rigorous attitude of scholarship and research integrity, cautiously grasp current controversial research directions, and carefully study and judge possible ethical and moral risks of biomedical technology to minimize harm, but also an open strategy of dynamic adjustment and regular revision of identification criteria and scope definition. For example, China’s forthcoming *Regulations on the Safety Management of Biotechnology Research and Development (Draft for Public Comments)* states that “a list of prohibited biotechnology research and development activities shall be formulated, dynamically evaluated, revised at an appropriate time, and promptly released.” [54] This approach can highlight management focus, clarify the main objectives of risk prevention and control, narrow the scope of regulation, and balance national security and public interest as much as possible through management and promotion. It aims to promote the healthy and sustainable development of biomedicine through policy guidance.

Third is the principle of responsibility. In contrast to nuclear technology, in the life sciences, autonomy of the relevant stakeholders is an important feature [55]. The key to governance lies in humans, and the core of integrating development and safety must be internalized by humans. Life scientists are on the front line of biomedical technology development, and are the first line of defense against the misuse and misapplication of technology. Raising the awareness of the safety responsibility of scientists, strengthening ethical self-discipline, and adhering to the principle of bold research and prudent promotion are the endogenous forces that prevent the misuse of biomedical technology while realizing sustainable development. Based on the principle of responsibility in the right to science, in addition to requiring practitioners to proactively comply with the laws on research, and to resist illegal behavior circumventing regulation, subsequent legislation must also clearly and comprehensively make the entities researching, developing, and applying biomedical technology legally responsible. In particular, the main responsibilities of medical institutions conducting clinical research should be clearly defined, and the penalties for violating the law should be enforceable, and increased. In addition, to enhance the authority and implementation of integrated management of biomedical technology risks, the relevant laws and regulations should also provide a system of rewards and punishments: entities and individuals who are meritorious in denouncing the misuse and abuse of biomedical technology should be given commendations and rewards, while those who violate the law to carry out research and clinical development and application should be given clear penalties such as warnings, limited time for corrections, fines, expulsion or dismissal, and lifetime bans on research. If the situation is serious, criminal liability should also be pursued. Offending entities should be notified and criticized, or ordered to make corrections within a certain period; if the circumstances are serious, administrative sanctions should be imposed on the supervisors and directly responsible persons by law.

### 4.2 Impact on legal practices

Further legislation also endorses the right to science (See below Table 2). Both the *Popularization of Science & Technology Act* and the *Science & Technology Progress Act* underscore the national duty to promote and safeguard research. These laws manifest the state’s commitment to safeguarding the legitimate rights and interests of scientists, concurrently accentuating the freedom to engage in research [56], [57]. In these textual formulations, the Chinese government underscores one facet of the right to science, namely the freedom to engage in research. Nevertheless, it does not specifically mention participants or considerations related to the right to benefit from involvement in scientific discoveries. Despite this, the emphasis is directed towards actualization of the right to science, giving particular attention to compensating scientists and ensuring their entitlement to benefits derived from their research. The Ministry of Science and Technology (MoST) emerges as the entity most intimately associated with the right to science. It is primarily focused on formulating development strategies and regulations for science and technology in a broad sense, including biotechnology. The aim is to address conflicts that may arise in the intersection between technology and socioeconomic development [58].

**Table 2:** Practical legal effects of the “right of science on China’s medical biotechnology policy.

Impact of the "Right to Science" on China’s Medical Biotechnology Policy		
	Freedom supported by the “right to science”	Limitations set by the “right to science”
**The Impact on Legal Practices**		
*Law of the People's Republic of China on Popularization of Science & Technology*	**Article 5** requires the government to protect PST organizations and workers, endorsing independent activities and lawful initiation of research.	
*Scientific and Technological Progress Law of the People's Republic of China*	**Article 3** ensures freedom of research, encourages innovation, and protects scientists' and technicians’ rights and interests.	
*Law of the People's Republic of China on the Progress of Science and Technology*	**Article 8** stipulates freedom of technological research, promotes exploration and innovation, and protects the rights of science and technology personnel. **Chapters III and IV:** Enterprise-driven research, innovation, and practical application of research.	**Article 15** stipulates that the State Council oversees national science & technology progress and integrates it into economic plans; local governments enhance the organization and management. **Chapters X and XI:** Supervision and Legal Liability
*Biosecurity Law of the People’s Republic of China*	**Chapter II:** Risk Control Systems (aims to harness the contradictions between freedom and its limitations in the context of the “right to science”)	**Article 1** stipulates this law aims to ensure national security, address biosecurity risks, and promote the harmonious coexistence of humans and nature. **Chapter II:** Risk Control Systems **Article 11** stipulates that the National Biosecurity Coordination Mechanism integrates key government departments, analyzing trends and coordinating efforts.

The recent *Law of the People’s Republic of China on Progress in Science & Technology* is a noteworthy instance of high-level legal text uniquely designated after the legislation within the jurisdiction of judicial sovereignty [11]. This law specifically addresses and pertains directly to technology. Evident in the text is China’s endorsement of domestic financial backing for advancement of scientific research. Simultaneously, it carefully evaluates constraints on scientific progress, resembling the more rigorous limitations imposed by international human rights legislation in various global jurisdictions. The guiding principles articulated in this text, emphasizing encouragement, support, and protection, encapsulate the nation’s standpoint on liberty of scientific inquiry. Moreover, as elucidated in the preamble, this freedom is substantially fortified, extending in reach from individual citizens to societal institutions [11]. This legislation explicitly confers authority in the realm of technological progress. Governments at or above the county level are mandated to incorporate technology initiatives into their social governance planning, with the State Council designated as the national leader in this domain [11]. It is evident that researchers constitute a crucial beneficiary of the right to science, as articulated in this recently enacted legislation. The provisions explicitly recognize academic freedom as falling within the sovereign jurisdiction of a nation. Moreover, the third and fourth chapters delineate specific regulations concerning the actualization of technological achievements, commercialization of intellectual property, and technological innovation within private enterprises [11]. Intriguingly, this regulation maintains administrative coherence concerning researchers and the benefits derived from their research by delineating management authority into two tiers. The State Council is entrusted with policy formulation and setting the overarching direction, while local governments at the county level or above are tasked with executing specific responsibilities related to fostering a favorable market environment. As a result, Chinese law expressly acknowledges the legal standing of researchers about another facet of the right to science, specifically regarding enjoyment of benefits and welfare arising from technological advancements. Chapters 10 and 11 predominantly delineate the rights and constraints on freedom within the right to science [11]. Concurrently, the *Law* also establishes a Technological Ethics Committee, elucidating the powers and penalties at the disposal of units and relevant supervisory bodies of scientific and technological personnel when confronted with transgressions related to integrity, technological ethics, harm to public interests and national security, or infringements on human health during their research. Its judicious use of restraint and moderate ambiguity is considered an appropriate legislative technique, taking into account the hierarchical nature of Chinese law and the fact that it is a high-level legal document enacted by the National People's Congress. While the *Law* refrains from providing explicit definitions of scientific ethics or detailed evaluation mechanisms, this approach aligns with the appropriate Chinese legislative norms.

Another prominent law in the field of technology which has garnered widespread attention in recent years is the *Biosecurity Law of the People’s Republic of China*, enacted in 2020 [12]. The preamble, outlines the purpose of the *Law*, to safeguard the health of the public and protect biological resources and the environment [12]. The second chapter establishes a comprehensive biosafety risk prevention and control system, ranging from a biosafety risk monitoring and early warning systems to biosafety catalogs and lists, as well as a biosafety review system. It also introduces multi-level risk classification [12]. Notably, similar to the *Law of the People’s Republic of China on Progress of Science & Technology*, in this law, the State Council serves as the primary leadership authority, while local governments at various levels are the actual implementers responsible for the tasks at hand. From these high-level legal documents, we can discern a development in China’s legislative practice: cross-referencing of multiple legal documents and unclear delineation of functions among different institutions with shared regulatory authority is gradually being clarified, with power consolidating under the central government for unified leadership. Consequently, the previously unclear status of scientists regarding management and regulation of their conduct or freedoms is being addressed. While power is consolidated under the unified leadership of the State Council, as mentioned in the *Biosecurity Law*, national biological security work will involve health, agriculture, rural areas, science & technology, and relevant military agencies [12]. Therefore, the precise details of a working model—whether it should be led by an existing administrative department with support from other departments or if relevant departments should integrate personnel to establish a new department— require further dedicated efforts.

The two most influential legal frameworks in China on the balance between risk and innovation in medical biotechnology explicitly emphasize the right to science, and in particular the freedom to conduct research. Although no direct legal provisions explicitly state the aspects of freedom related to the benefits derived from participation in science, its emphasis is discernible through their protection of the rights of participants, the state’s protection of the rights of citizens, and support for various aspects of social life. When it comes to the *Right to Science*, the relevant statutory provisions emphasize adherence to technical ethics, with some prohibitions specifically protecting human dignity. Nevertheless, clear and detailed definitions, as well as refined evaluation criteria, are not directly discernible. In addition, regarding freedoms, Chinese law outlines a broad framework for technological development and institutional safeguards within the “right to science,” but the precise details within this framework remain ambiguous. Going through all the sections and provisions of the *Biosecurity Law*, compared with the *Law on the Advancement of Science & Technology*, reveals a remarkable distinction. The former focuses on how the state uses its public authority to ensure that technological development operates consistently within public acceptance, while the latter focuses on legally empowering research entities with the rights they need to engage in research. Neither law addresses how to balance conflicting rights of different entities within their respective domains. The Chinese legal framework seems to conceptualize the right to science not merely as a concept within its human rights system that adjusts the adversarial relationships between private rights holders. The expression of the right to enjoy scientific development remains subdued. One might infer that the legislature does not perceive the enjoyment of the benefits and freedoms derived from technological progress and the conduct of scientific research within this framework as concepts prone to formation of adversarial relationships. Perhaps this is due to the paramount role of uniform regulation in this domain of judicial sovereignty: the executive, elected by the people and representing the entire population, supports technological development on behalf of the nation, regulating and restricting technology. In this model, technology becomes an object of governance, and all citizens stand together to collectively experience its freedom and benefits, despite their diverse identities.

## 5 Discussion and conclusion

This paper provides a comprehensive picture of the impact of the “right to science” on China’s medical biotechnology regulatory policy by analyzing China's attempts to maintain a sustainable balance between innovation and risk in boths legislative principles and practices. We discuss and assess the development of the right to science in Europe and the U.S., as well as the context of China’s efforts to respond to the changing external environment. Establishment of a legal right to science in China has lagged, and the He Jiankui incident cannot be ruled out in accelerating Chinese policies regarding management of technology. However, as a developing nation, China’s historical development in technology has been a prolonged and solitary journey, in which it has faced the challenge of seeking technological breakthroughs within the core technological constraints imposed by developed nations. As a third-world country and a prominent player in science and technology, competing with the EU and the U.S. in terms of funding and research, China seems to have developed a distinctive legal model for technological governance. China has charted a new course, unlike the EU's cautious approach toward new technological risks, or the U.S. emphasis on encouraging technological innovation while downplaying regulation. Comparative analysis on the theoretical causes of policy differences between Europe and the U.S. favoring either precaution or innovation has revealed that China’s legal framework possesses unique characteristics of considerable value.

China’s strategic and method outlook differ from those of the West, as the cultural characteristics of Confucianism permeate the fabric of its legal construction. On the one hand, it lacks the multi-party governance system prevalent in Europe and the U.S. On the other hand, issues related to populist tendencies favoring either precaution or innovation, as well as concerns regarding technological bureaucracy, find no practical root in China’s political system. The legal framework associated with the right to science, particularly the technological monitoring and regulatory system, does not flourish in a political landscape characterized by party confrontations, or within a sovereignty domain where party transitions result in discontinuity of governance. While the ruling party is obligated to consider the will of citizens across different historical stages, its motives are not necessarily pure and untainted; often, they are entwined with the prospect of securing votes in the next electoral cycle. However, the risks posed by technological developments do not limit themselves to a specific aspect of the world based on party or political preferences. These risks typically affect society as a whole, analogous to the impacts of environmental contamination. China’s single-party political system prevents discontinuity in legal systems due to party confrontation. Therefore, as a rapidly developing economy in science and technology, China’s exploration of the balance between risk and innovation may possess institutional and objective advantages that the debate-ridden European and American systems do not. In other words, the impact of the right to science on the policies regulating emerging technologies, including biomedical, may find the most suitable development in China. We believe that China’s medical biotechnology regulatory policy still has a long way to go in balancing risk and innovation. It is vital to get these policies right, not only for China but also to inspire policy development in other countries with growing life science and medical biotechnology sectors. Balancing the need for innovation with measures to ensure that work is done safely, securely, and responsibly is a task for each government.

## Author statement

Y.H. and Y.X. developed the argument presented in this article. Conceptualization, Y.H., and Y.X.; Methodology, Y.H., literature review, Y.H., L.F., and Y.X.; writing—original draft preparation, Y.H., and Y.X.; writing—review and editing, Y.H., and Y.X.; supervision, Y.X.; project administration, Y.X. All authors have read and agreed to the published version of the manuscript.

## CRediT authorship contribution statement

**Lindsay L Fan:** Formal analysis, Writing – original draft, Investigation. **Yiping Han:** Conceptualization, Formal analysis, Methodology, Writing – original draft, Writing – review & editing. **Yang Xue:** Conceptualization, Formal analysis, Investigation, Project administration, Supervision, Writing – original draft, Writing – review & editing.

## Declaration of Competing Interest

The authors declare no conflicts of interest.
